# Suppression of cognitive function in hyperthermia; From the viewpoint of executive and inhibitive cognitive processing

**DOI:** 10.1038/srep43528

**Published:** 2017-03-07

**Authors:** Manabu Shibasaki, Mari Namba, Misaki Oshiro, Ryusuke Kakigi, Hiroki Nakata

**Affiliations:** 1 Department of Health Sciences, Faculty of Human Life and Environment, Nara Women’s University, Nara, Japan; 2 Graduate School of Humanities and Sciences, Nara Women’s University, Nara, Japan; 3 Department of Integrative Physiology, National Institute for Physiological Sciences, Okazaki, Japan

## Abstract

Climate change has had a widespread impact on humans and natural systems. Heat stroke is a life-threatening condition in severe environments. The execution or inhibition of decision making is critical for survival in a hot environment. We hypothesized that, even with mild heat stress, not only executive processing, but also inhibitory processing may be impaired, and investigated the effectiveness of body cooling approaches on these processes using the Go/No-go task with electroencephalographic event-related potentials. Passive heat stress increased esophageal temperature (Tes) by 1.30 ± 0.24 °C and decreased cerebral perfusion and thermal comfort. Mild heat stress reduced the amplitudes of the Go-P300 component (i.e. execution) and No-go-P300 component (i.e. inhibition). Cerebral perfusion and thermal comfort recovered following face/head cooling, however, the amplitudes of the Go-P300 and No-go-P300 components remained reduced. During whole-body cooling, the amplitude of the Go-P300 component returned to the pre-heat baseline, whereas that of the No-go-P300 component remained reduced. These results suggest that local cooling of the face and head does not restore impaired cognitive processing during mild heat stress, and response inhibition remains impaired despite the return to normothermia.

Climate change has had a widespread impact on humans and natural systems^1^,^2^. In recent decades, a number of severe heat waves have occurred throughout the Northern Hemisphere^3^. By 2050, it has been estimated that many US cities will more frequently experience extremely hot days^4^. The frequent occurrence of heat waves or the urban heat island phenomenon poses a significant threat to human health^5^. Heat stroke is a life-threatening condition that is characterized by an elevated core body temperature that increases to more than 40 °C and dysfunctions in the central nervous system^6^.

Prior to the development of heat stroke, signs including fatigue, weakness, dizziness, and decreased consciousness are observed with long-term exposure to a hot environment. Cognitive function may subjectively decrease with hyperthermia, however, the effects of hyperthermia on cognitive function remain equivocal because of methodological discrepancies including research approaches as well as environmental and physiological conditions^7^. Cognitive function mainly involves two broad questions: how knowledge is represented in the brain and how behavior is controlled. In the latter, motor inhibition is the essential skill for adapted behavior requiring motor control. In the process of decision-making, executive and inhibitory functions are coordinated in high-order functions. However, difficulties are associated with evaluating these stop actions because the underlying processes of inhibition are not directly observable. Electroencephalographic event-related potentials (ERPs) using the Go/No-go task are a useful paradigm for investigating executive and inhibitory processes. The primary purpose of the present study was to assess the effects of heat stress on the cognitive processing of executive and inhibitory responses.

One of the potential mechanisms that clinically alter cerebral hemodynamics is related to cognitive decrements^8^,^9^. Heat stress acutely decreases cerebral perfusion when core temperature increases by more than approximately 1.2 °C^10^,^11^,^12^. Reduced cerebral perfusion as well as increased brain temperature may contribute to impairments in the brain neural network and cognitive processing. In a hot environment, cooling the face and/or head is a natural behavior for improving thermal comfort and arousal. Cold or cool stimuli to the body surface are perceived as being comfortable and increase alertness when body temperature is high^13^,^14^. Based upon these natural behaviors, coupled with heat stress increasing facial and head skin blood flow for heat dissipation^15^, facial and head cooling may restore brain function as well as cerebral perfusion. However, the effectiveness of these natural behaviors on cognitive function and/or cerebral perfusion has not yet been investigated.

We hypothesized that heat stress impairs cognitive inhibitory and executive processing, while the restoration of cerebral perfusion and/or thermal comfort by facial and head cooling may lead to the recovery of cognitive processing. Alternatively, increased brain temperature itself may impair cognitive processing. In order to test these hypotheses, subjects performed four sessions of the Go/No-go tasks: rest (1^st^ session), during heat stress (2^nd^ session), during heat stress with facial and head cooling (3^rd^ session), and whole body cooling after heat stress (4^th^ session).

## Results

### Temperature variables

Approximately 60 minutes of whole body heating by circulating hot water through a tube-lined suit increased mean skin temperature and esophageal temperature (Tes) by 4.66 ± 0.63 °C and 1.30 ± 0.24 °C, respectively. In order to maintain Tes during the Go/No-go tasks in the two sessions (2^nd^ and 3^rd^ sessions), circulating water temperature was controlled. During the 2^nd^ session of the Go/No-go task, Tes slightly increased (37.98 ± 0.21 °C to 38.02 ± 0.19 °C, measured at the onset and end of the 2^nd^ session), however, these changes were within 0.1 °C (averaged data during the task shown in Table 1). After the 2^nd^ session, facial and head cooling (FHC) was applied. Tes was slightly decreased (37.98 ± 0.19 °C) before the start of the 3^rd^ session, but was the same as that of the start of the 2^nd^ session. At the end of the 3^rd^ session, Tes was 37.89 ± 0.15 °C, however, these changes were also within 0.1 °C. Tes during the 3^rd^ session was maintained at +1.24 ± 0.22 °C above the rest temperature (i.e. before heat stress). After FHC, cold water was circulated through the tube-lined suit in order to decrease body temperatures (whole body cooling, WBC). The 4^th^ session was performed when Tes decreased by 0.5 °C or more from the end of the 3^rd^ session.

### Blood flow variables

Internal carotid artery (ICA) blood flow was reduced during whole body heating, while common carotid artery (CCA) blood flow, estimated external carotid artery (ECA) blood flow (by subtracting blood flow in the ICA from that in the CCA), and cutaneous vascular conductance (CVC) (the ratio of skin blood flux to mean arterial blood pressure) at the forehead and forearm were increased. Following the 2^nd^ session, face and head cooling significantly decreased ECA blood flow and CVC at the forehead as well as local skin temperature at the forehead, and ICA blood flow returned to the pre-heat level.

### Behavioral data

**Figure 1:**
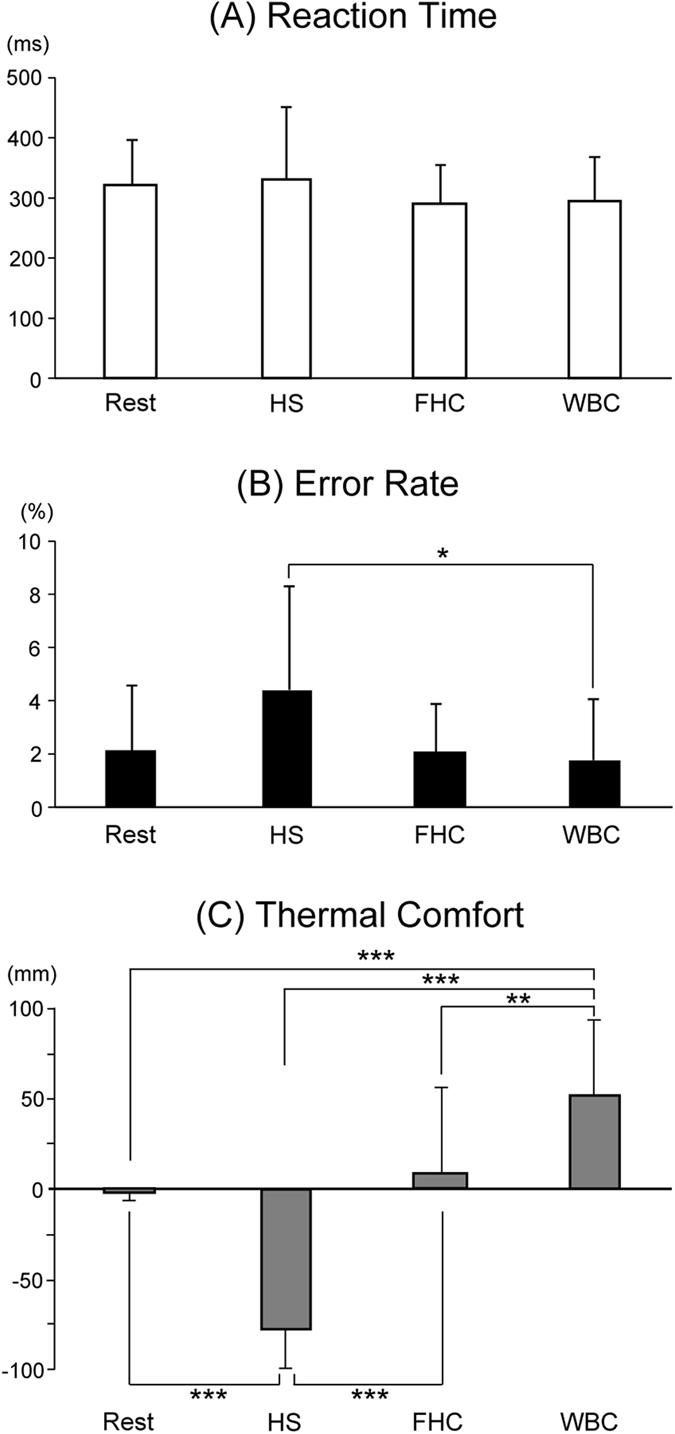
Mean reaction time, error rate, and thermal comfort. Reaction times did not change throughout sessions, whereas a significant main effect among Sessions was observed in the error rate and thermal comfort. Post-hoc tests revealed that the error rate was significantly greater in HS than in WBC (*p < 0.05), while no significant difference was observed between Rest and other sessions. Thermal comfort was significantly reduced by heat stress (HS), but restored during face/head cooling (FHC), while subjects felt thermally comfortable during whole body cooling (WBC) (**p < 0.01; ***p < 0.001). Values are the mean ± standard deviation.

Figure 1A shows mean reaction times. No significant main effect of Session was observed. Figure 1B shows the error rate, with a significant main effect of Session being observed (F (3, 42) = 4.239, p < 0.05). Post-hoc tests demonstrated that the error rate was significantly larger during HS than during WBC (p < 0.05), while no significant difference was observed between Rest and other sessions. Figure 1C shows thermal comfort with SD, with a significant main effect of Session being observed (F (3, 42) = 47.986, p < 0.001). Post-hoc tests demonstrated that thermal comfort was significantly higher during WBC than during Rest, HS, and FHC (p < 0.001, p < 0.001, and p < 0.01, respectively), and also during Rest and FHC than during HS (p < 0.001, respectively).

### Amplitude and latency of the P300 component

**Figure 2:**
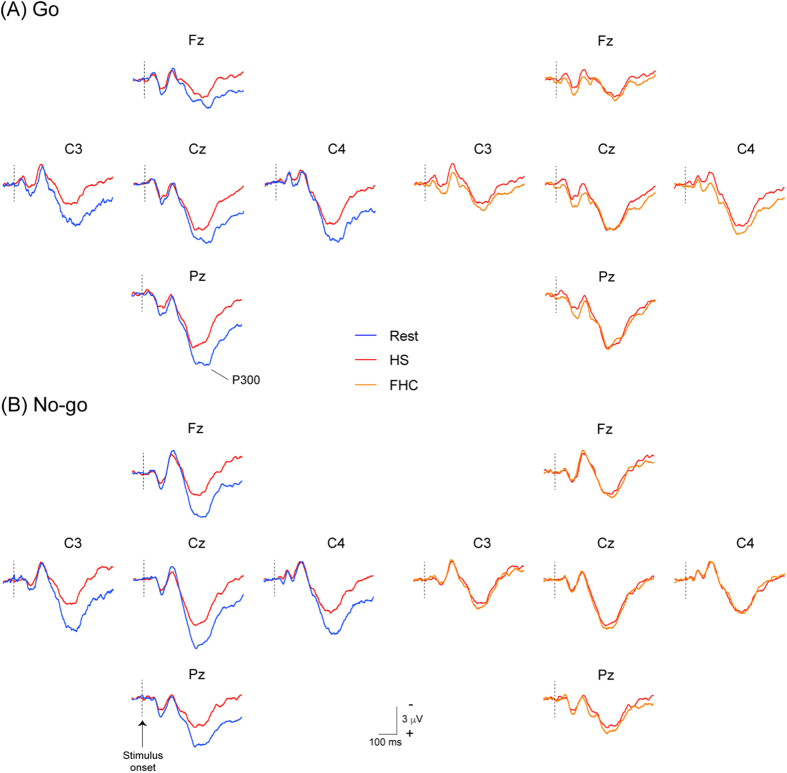
Grand-averaged event-related potentials (ERP) waveforms at five sites (Fz, Cz, Pz, C3, and C4) between the pre-heat baseline (1^st^ session, Rest), during heat stress (3^rd^ session, HS), and during heat stress with face/head cooling (3^rd^ session, FHC) in Go trials (Panel A, upper figures) and No-go trials (Panel B, lower figures). Blue, red, and orange lines show waveforms in the 1^st^, 2^nd^, and 3^rd^ sessions, respectively. The amplitudes of the P300 component in both trials are clearly reduced during HS and remain reduced during FBC.

**Figure 3:**
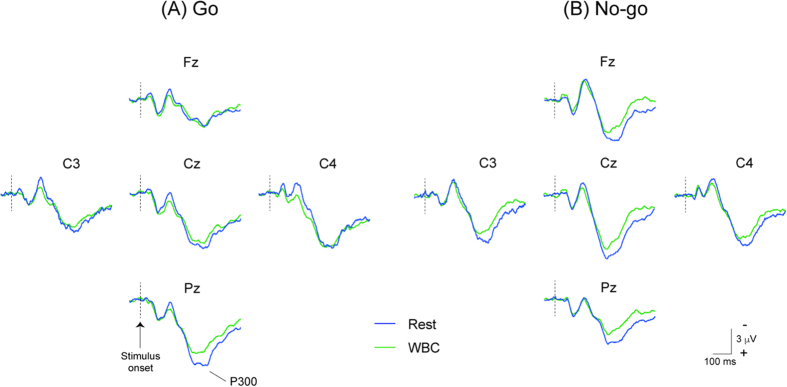
Grand-averaged event-related potentials (ERP) waveforms at five sites (Fz, Cz, Pz, C3, and C4) between the pre-heating baseline (1^st^ session, Rest) and during whole body cooling (4^th^ session, WBC) in Go trials (Panel A, upper figures) and No-go trials (Panel B, lower figures). Blue and green lines show waveforms in the 1^st^ and 4^th^ sessions, respectively. The amplitudes of the P300 component in the Go trials are similar between the pre-heat baseline (1^st^ session, Rest) and recovery (4^th^ session, WBC), except for Pz, whereas those in the No-go trials remain smaller during WBC than during Rest.

**Figure 4:**
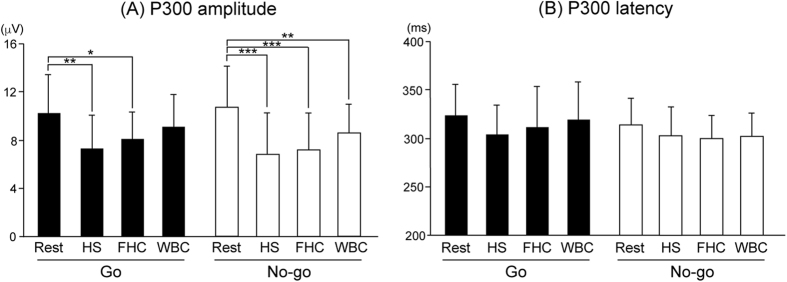
The mean P300 peak amplitude (**A**) and latency (**B**) in Go trials (left) and No-go trials (right) averaged across all electrodes. Post-hoc testing demonstrated that the amplitude of Go-P300 was significantly reduced during heat stress (HS) and face/head cooling (FHC), but recovered during whole body cooling (WBC), whereas the amplitude of No-go-P300 was significantly reduced during HS, FHC, and WBC. *p < 0.05, **p < 0.01, ***p < 0.001 from Rest. Values are the mean ± standard deviation.

Electroencephalographic ERPs were recorded at Fz, Cz, Pz, C3, and C4, according to the International 10–20 System. Figures 2 and 3 show the grand-averaged waveforms of P300. The amplitude of Go-P300 was similar between Rest and WBC at four electrodes, except for Pz, while the amplitude of No-go-P300 was markedly smaller during WBC than during Rest at all electrodes. ANOVAs for amplitude showed the significant main effect of Session (F (3, 42) = 10.701, p < 0.001). Post-hoc testing demonstrated that the amplitude of Go-P300 was significantly smaller during HS and FHC than during Rest (p < 0.01 and p < 0.05, respectively), while that of No-go-P300 was significantly smaller during HS, FHC, and WBC than during Rest (p < 0.001, p < 0.001, and p < 0.01, respectively) (Fig. 4A). ANOVAs for the peak latency of the P300 component revealed no significant main effect or interaction (Fig. 4B).

## Discussion

We herein demonstrated that the neural activities of response execution (i.e. amplitude of the Go-P300 component) and response inhibition (i.e. amplitude of the No-Go P300 component) were decreased when Tes increased by ~1.2 °C. FHC returned cerebral blood flow and thermal comfort to pre-heat baseline levels, whereas executive and inhibitory processing both remained reduced. WBC following mild hyperthermic challenges markedly decreased temperatures at all the body parts measured. WBC recovered executive processing, whereas inhibitory processing remained reduced.

Core executive functions are inhibition including response inhibition and interference control (selective attention), working memory, and cognitive flexibility^16^. Impairments induced in cognitive performance during heat stress have been suggested to depend on the complexity and/or duration of cognitive tasks^17^,^18^,^19^,^20^ as well as the severity and duration of heat stress^21^. We previously demonstrated, even in ‘simple’ auditory oddball tasks, that hyperthermia reduced the cognitive processing index (i.e. the amplitude of P300) during “severe” heat stress^22^. P300 induced by an auditory oddball task was proportional to the amount of “attentional resources” (i.e. selective attention) because of the occurrence of an “infrequent” target stimulus^23^,^24^. In the present study, we focused on the effects of heat stress on “response inhibition”. Human motor control consists of response execution and response inhibition, and the somatosensory Go/No-go task is useful for investigating inhibitory function (by the amplitude of No-go-P300). Moreover, the prefrontal cortex is widely considered to play a key role in supporting cognitive function, particularly inhibitory function^25^. The anteriorization of No-go-P300 has frequently been recorded; No-go-P300 shows a more anterior distribution than Go-P300 and classical P300^26^,^27^,^28^. We concurrently performed this study during moderate heat stress (a core temperature increase of 1.0 to 1.5 °C) because this level of an increase in core temperature decreases cerebral perfusion and increases thermal discomfort. As described above, response inhibition as well as response execution were reduced during mild heat stress, and neither recovered with the application of FHC. WBC recovered response execution, but not response inhibition. These differences may be related to the neural network and/or regional differences in brain activity.

The neural network associated with executive and inhibitory processing has been examined using functional magnetic resonance imaging (fMRI), and these processes include the dorsolateral (DLPFC) and ventrolateral prefrontal cortices (VLPFC), supplementary motor area (SMA), primary sensorimotor area (SMI), anterior cingulate cortex (ACC), temporoparietal junction, temporal and parietal lobes, and thalamus^29^,^30^,^31^,^32^,^33^,^34^. Thus, increases in brain temperature may reduce neural activities in these regions because Tes was maintained during HS and FHC, whereas cerebral perfusion was returned to the pre-heat level during FHC. Furthermore, our previous findings, which were obtained using the somatosensory Go/No-go task, showed that the strength of neural activity was greater in No-go trials than in Go trials at the DLPFC, VLPFC, ACC, inferior parietal lobule, and caudate^34^. Therefore, these areas may be related to the long-lasting effects of elevated brain temperature because Tes returned almost to normothermia during WBC. Alternatively, the neural network of cognitive processing may have become obstructed when brain temperature increased over a given level. In our previous study^35^, we also investigated the temporal dynamics of neural activation for somatosensory Go/No-go trials, and found that activated regions and timing in the primary somatosensory cortex, secondary somatosensory cortex, and SMA were similar between the Go and No-go trials. The premotor area (PM) was subsequently activated in Go trials, while the PFC was activated in No-go trials. The ACC was activated in both trials. We speculate that if elevated brain temperature obstructs the neural network, the PFC may be associated with the long-lasting effects of elevated brain temperature. Further studies are needed in order to clarify the brain regions responsible for this effect in hyperthermic individuals.

Mild hyperthermia reduces cerebral perfusion^5^,^21^, indicating that restricted cerebral perfusion contributes to an impaired brain neural network and reduced cognitive processing. We previously demonstrated that passive heat stress decreased ICA blood flow, but increased ECA for heat dissipation^15^. Therefore, we attempted to recover ICA to the pre-baseline level by cooling the face and head in the present study. Consistent with previous findings, ICA blood flow was decreased by HS, and was returned to the pre-baseline level by FHC. However, executive and inhibitory processing evaluated by electroencephalographic ERPs did not recover during FHC. Therefore, restricted cerebral perfusion might contribute to an impaired brain neural network and reduced cognitive processing. Rasmussen *et al*. previously reported that reduced alertness evaluated by the electroencephalographic power spectrum during hyperthermia was not associated with decreased cerebral blood flow^36^. Therefore, increased brain temperature itself contributes to reduced cognitive processing. Hyperthermia decreases cerebral metabolism in the caudate, putamen, insula, and posterior cingulum^37^, which may obstruct the cognitive neural network. The insula has numerous neural connections, including the cingulate cortex, caudate putamen, and thalamus^38^. Taken together, our results and previous findings showed that elevations in brain temperature due to HS decreased cerebral metabolism, which may impair cognitive processing.

In conclusion, the present study demonstrated that executive and inhibitory processing are both impaired when core temperature increases to ~38.0 °C. Cooling the face and head may recover thermal comfort and cerebral perfusion. However, cognitive processing remains impaired when core temperature is elevated. Decreases in body temperature restore response execution, whereas the effects of hyperthermia on inhibitory processing persist.

## Methods

Fifteen male subjects participated in this study. The age, body mass, and height of the subjects were 21 ± 1 yrs, 73.2 ± 14.2 kg, and 172.2 ± 6.2 cm, respectively. All subjects were informed of the study protocol and risks before providing their written informed consent. This study was approved by the Ethics Committee of Nara Women’s University, Nara, Japan. The protocol was performed in accordance with the Declaration of Helsinki.

### Experimental Procedures

Subjects arrived at a temperature-controlled laboratory (26 ± 1 °C) at least 2 h after a light meal. Following nude body mass measurements, a thermocouple was inserted into the nasal passage to a distance equivalent to one-fourth of the subject’s height in order to measure Tes and six thermocouples were attached to the skin (chest, abdomen, upper and lower back, thigh, and calf) to obtain Tsk. Tsk was calculated from the weighted average of the six points^39^. Each subject, wearing only underwear and short pants, was dressed in a tube-lined water-perfused suit (Med-Eng, Ottawa, Ontario, Canada). The water-perfused suit covered the entire body, except for the head, face, hands, and feet. Subjects rested quietly in the semi-supine position (the upper body was tilted by 15°) for at least 30 min while normothermic water (33 °C) perfused the suit, thereby maintaining thermoneutral conditions. Other instruments were attached during the equilibration period. Subjects also received instructions regarding the Go/No-go task (see the description below) and practiced it several times (~20 trials). Subjects were instructed how to use the visual analogue scale (VAS) to score thermal comfort. The first Go/No-go task (pre-heat session) was performed while the subject was in a normothermic condition. Passive heat stress was initiated by perfusing 50 °C water through the suit. Approximately 60 min after initiating hot water circulation, when Tes approached 1.1 °C, water temperature was slightly reduced to attenuate the rate of the increase in Tes during Go/No-go tasks. The 2^nd^ Go/No-go task was performed after Tes increased by ~1.2 °C from the pre-heat stress baseline (HS session). After the Go/No-go task, subjects’ faces were thermally cooled using a fan (~0.5 m from their face) and an ice pack was placed under their occipital region while whole body heating was continued. Five minutes after initiating this cooling, the 3^rd^ Go/No-go task was performed (FHC session). While performing both trials, water temperature was regulated to maintain the core temperature. Cold water (25 °C) was immediately perfused through the suit to decrease Tsk. When Tes decreased to ~37.0 °C, each subject performed the 4^th^ Go/No-go task (WBC session).

### The somatosensory Go/No-Go task

The Go stimulus was delivered to the second digit of the left hand, and the No-go stimulus to the fifth digit of the left hand. Subjects had to respond to the stimulus by pushing a button with their right thumb (contralateral to the stimulated side) as quickly as possible only after presentation of the Go stimulus. Electrical stimuli were applied to the second or fifth digit of the left hand with ring electrodes. The electrical stimulus used was a current constant square-wave pulse that was 0.2 msec in duration, and the stimulus intensity was 2.5-fold that of the sensory threshold, which yielded no pain or unpleasant sensations. The probability of the stimulus for the second and fifth digit was even. Stimuli were presented in a random order, with the interval of presentation being fixed at 2 sec. The reaction time was measured for the Go stimulus. Each session comprised 80 epochs of stimulation, which included 40 epochs for the Go stimulus and 40 for the No-go stimulus. Subjects kept their eyes open and focused on a small fixation point positioned in front of them at a distance of approximately 1 m throughout each task. The error rate, which included commission (i.e. error pushing in No-go trials) and omission errors (i.e. slow response or no pushing in Go trials), was calculated in each session.

### Instrumentation and measurements

#### Electroencephalogram (EEG recordings)

EEGs were recorded with Ag/AgCl disk electrodes placed on the scalp at Fz, Cz, Pz, C3, and C4, according to the International 10–20 System. Each scalp electrode was referenced to linked earlobes. The ground electrode was placed at Fpz. In order to aid in the elimination of data during eye movements or blinks, an electro-oculogram was recorded bipolarly with a pair of electrodes placed 2 cm lateral to the lateral canthus of the right eye and 2 cm above the upper edge of the right orbit. Impedance was maintained at less than 5 kohm and was confirmed before each session. If impedance exceeded 5 kohm, the electrode was repasted at that location and the correct impedance confirmed. EEG signals were collected on a signal processor (Neuropack MEB-2200 system, Nihon-Kohden, Tokyo, Japan). The analysis epoch for each ERP stimulus was 600 msec, including a pre-stimulus baseline period of 60 msec. The band-pass filter was set to 0.1–50 Hz and the sampling rate was 1000 Hz. No digital filter was applied off-line. The peak amplitudes and latencies of the P300 component of EEG were measured at 240–500 msec, respectively. If the P300 component showed double peaks, we took the value from the largest peak. Amplitudes were measured baseline-to-peak. Slow responses exceeding 500 msec and incorrect responses were eliminated from averaging.

#### Thermoregulatory and hemodynamic variables

In addition to the aforementioned Tes and Tsk, Tear was measured using an infrared sensor (Nipro CE Thermo, NIPRO, Japan), which was insulated with cotton and covered by cling film. Heart rate was obtained from an electrocardiogram (Biomulti 1000, NEC, Tokyo, Japan) and intermittent arterial blood pressure by auscultation of the brachial artery via electrosphygmomanometry (STBP-780, Colin, Tokyo, Japan). Skin blood flux was measured via laser-Doppler flowmetry (moor VMS-LDF2, Moor Instruments, UK) using a combined temperature and 8 collecting fibers-bundled probe (VP1T/7, Moor Instruments, UK) attached to the forehead and left forearm. These variables, except for blood pressure, were continuously measured and sampled at 20 Hz via a data acquisition system (MP150, BIOPAC Systems, Santa Barbara, CA, USA), while blood pressure was obtained before each Go/No-Go task. CVC was calculated from the ratio of skin blood flux to mean arterial blood pressure. Blood flow was measured in the left ICA and CCA using a color-coded ultrasound system (Vivid-i; GE Healthcare, Tokyo, Japan) equipped with a 10 MHz linear transducer. ICA and CCA blood flow measurements were performed ~1.0–1.5 cm distal and proximal to the carotid bifurcation, respectively. Blood flow was measured 1 min before each session. ECA blood flow was estimated by subtracting blood flow in the ICA from that in the CCA. The diameters of each vessel were measured at three points in a longitudinal section using the brightness mode, and the Doppler velocity spectrum was subsequently identified by the pulsed wave mode. Systolic and diastolic diameters were measured in detail, and the mean diameter (cm) was then calculated in relation to the blood pressure curve: mean diameter = [(systolic diameter × 1/3)] + [(diastolic diameter × 2/3)]. The time-averaged mean flow velocity obtained in the pulsed wave mode was defined as the mean blood flow velocity (cm/s). Blood flow velocity was measured from the average of ~10–20 cardiac cycles in order to eliminate the effects caused by the breathing cycle. When making blood flow velocity measurements, care was taken to ensure that the probe position was stable, that the insonation angle did not vary (~60 deg in most cases), and that the sample volume was positioned in the center of the vessel and adjusted to cover the width of the vessel diameter. Blood flow was calculated by multiplying the cross-sectional area [π × (mean diameter/2)^2^] by mean blood flow velocity; Blood flow = mean blood flow velocity × area × 60 (ml min^−1^). The same operator performed all blood flow measurements. The scores for thermal comfort VAS ranged between −100 mm (too hot and uncomfortable) and 100 mm (very comfortable), with a score of 0 mm indicating thermo-neutrality.

#### Data analysis

The peak amplitude and latency of the P300 component were analyzed by a three-way analysis of variance (ANOVA) with repeated measures using the within-subject factors of Stimulus (Go stimulus vs. No-Go stimulus), Session (1^st^, 2^nd^, 3^rd^, and 4^th^), and Electrodes (Fz, Cz, Pz, C3, and C4). In the presentation of figures, P300 responses were averaged across the five electrodes (i.e., Fz, Cz, Pz, C3, and C4), and were analyzed by a two-way ANOVA with repeated measures. Behavioral data (i.e. the mean reaction time and error rate) were analyzed by a one-way ANOVA with repeated measures using the within-subject factor of Session (1^st^,2^nd^,3^rd^, and 4^th^). Thermoregulatory and hemodynamic variables were also analyzed by a one-way ANOVA with repeated measures using the within-subject factor of Session. Mauchly’s sphericity assumption evaluation was performed for all repeated measures factors with more than two levels. A Greenhouse-Geisser adjustment was used if the results of that test were significant. When a significant main effect was identified, the Student-Newman-Keuls test was employed to identify specific differences. Statistical analyses were conducted using SPSS (Ver. 21). Significance was set at *p* < 0.05.

## Additional Information

**How to cite this article:** Shibasaki, M. *et al*. Suppression of cognitive function in hyperthermia; From the viewpoint of executive and inhibitive cognitive processing. *Sci. Rep.* **7**, 43528; doi: 10.1038/srep43528 (2017).

**Publisher's note:** Springer Nature remains neutral with regard to jurisdictional claims in published maps and institutional affiliations.

## Figures and Tables

**Table 1:** Thermoregulatory and hemodynamic variables.

	Rest (1^st^ session)	HS (2^nd^ session)	FHC (3^rd^ session)	WBC (4^th^ session)
Esophageal temperature, °C	36.71 ± 0.23	38.01 ± 0.20*	37.95 ± 0.18*	37.05 ± 0.21*†‡
External canal temperature, °C	36.98 ± 0.28	38.10 ± 0.34*	37.77 ± 0.32*	37.01 ± 0.42†‡
Mean skin temperature, °C	34.27 ± 0.63	38.93 ± 0.58*	38.53 ± 0.84*	34.34 ± 0.79†‡
Local skin temperature, °C				
Forehead	32.8 ± 1.1	34.5 ± 0.7*	31.3 ± 0.9*†	31.1 ± 1.5*†
Forearm	33.0 ± 0.9	36.9 ± 1.3*	36.8 ± 1.5*	33.2 ± 1.7†‡
CVC, au/mmHg				
Forehead	0.47 ± 0.17	1.53 ± 0.61*	1.42 ± 0.47*†	0.82 ± 0.52*†‡
Forearm	0.14 ± 0.12	0.90 ± 0.36*	0.88 ± 0.34*	0.53 ± 0.26*†‡
Heart rate, bpm	63.3 ± 7.3	105.4 ± 12.2*	102.8 ± 11.4*	73.4 ± 11.7*†
Mean arterial blood pressure, mmHg	91.7 ± 8.0	88.8 ± 7.7	87.4 ± 6.1	97.1 ± 8.8*†
Blood flow, ml/min				
Common carotid artery	506.5 ± 89.4	746.6 ± 131.7*	700.7 ± 156.4*	475.4 ± 79.7†‡
Internal carotid artery	356.9 ± 86.4	306.2 ± 74.5*	376.1 ± 118.2†	345.8 ± 107.2‡
External carotid artery (estimated)	149.6 ± 63.6	440.5 ± 80.1*	324.6 ± 107.5*†	129.7 ± 59.8†‡

HS; whole body heat stress, FHC; local face and head cooling during heat stress, WBC; whole body cooling (i.e. recovery).

CVC; cutaneous vascular conductance. Data except for blood flow measured by ultrasound were averaged during the task in each session.

Values are the mean ± SD. * P < 0.05 from the 1^st^ session; † from the 2^nd^ session; ‡ from the 3^rd^ session.
